# Plasma fibrinogen levels in women with infertility undergoing surgical evaluation for endometriosis: a retrospective cross-sectional study

**DOI:** 10.3389/fendo.2026.1929933

**Published:** 2026-09-10

**Authors:** Huanying Xu, Shaocheng Huang, Yuhan Zheng, Xue Mi, Yu Chen, Suzhen Wu

**Affiliations:** 1 Foshan Clinical Medical School of Guangzhou University of Chinese Medicine, Foshan, Guangdong, China; 2 Department of Traditional Chinese Medicine (TCM) Gynecology, Foshan Fosun Chancheng Hospital, Foshan, Guangdong, China; 3 Guangzhou University of Chinese Medicine, School of Basic Medical Sciences, Guangzhou, Guangdong, China

**Keywords:** coagulation, disease stage, endometriosis, fibrinogen, infertility, inflammation

## Abstract

**Background:**

Endometriosis is a chronic inflammatory disease linked to infertility. Coagulation-related processes may contribute to lesion persistence and pelvic remodeling, but the relevance of coagulation parameters in women with infertility undergoing surgical evaluation remains unclear. This study examined the association of plasma fibrinogen (FIB) with the presence and stage of endometriosis.

**Methods:**

This retrospective cross-sectional study included 389 women with infertility undergoing combined hysteroscopy and laparoscopy. Endometriosis was surgically confirmed and staged using the revised American Society for Reproductive Medicine classification. Plasma FIB was analyzed continuously and by quartiles. Multivariable logistic regression estimated odds ratios (ORs) and 95% confidence intervals (CIs) for overall and stage-specific endometriosis after adjustment for age, body mass index, education level, dysmenorrhea, and parity. Spline analyses explored potential nonlinearity. Activated partial thromboplastin time (APTT), prothrombin time (PT), thrombin time (TT), and international normalized ratio (INR) were exploratory comparators. Sensitivity analyses considered reproductive, inflammatory, and coagulation-related variables.

**Results:**

Among 389 women, 259 had endometriosis, including 186 with stage I–II and 73 with stage III–IV disease. Continuous FIB was not significantly associated with overall endometriosis (OR, 1.20; 95% CI, 0.76–1.91; P = 0.433). Compared with the first quartile, the third FIB quartile was associated with higher odds of overall endometriosis (OR, 2.37; 95% CI, 1.20–4.66; P = 0.012), with the largest estimate for stage III–IV disease (OR, 4.03; 95% CI, 1.56–10.43; P = 0.004). Q4 did not show a further increase, and trend tests were not significant. The adjusted spline increased across the lower-to-middle FIB range and then plateaued. The overall Q3 estimate remained elevated across sensitivity analyses, including the exploratory expanded model (OR, 2.27; 95% CI, 1.09–4.75). APTT, PT, TT, and INR showed no consistent associations.

**Conclusions:**

Plasma FIB did not show a simple linear association with endometriosis. A higher estimate was observed in the intermediate FIB range and persisted across several adjusted analyses, particularly in stage III–IV disease. This quartile-specific pattern remains exploratory and does not define a clinical cutoff. Independent cohorts are needed to determine whether this pattern is reproducible and clinically informative.

## 1 Introduction

Endometriosis is a chronic inflammatory disease characterized by endometrial-like lesions outside the uterine cavity and associated with pelvic pain, dysmenorrhea, dyspareunia, infertility, and reduced quality of life (1–3). In women with infertility undergoing surgical evaluation, laparoscopy visualizes lesion distribution, lesion size, and adhesions for diagnosis and staging; histopathology confirms the presence of endometrial-type tissue when specimens are obtained (4–6). Morphologic assessment alone does not reflect lesion-level coagulation activity or the accompanying microenvironmental context.

In endometriotic lesions, inflammation and cyclic bleeding interact with coagulation and fibrinolytic pathways. Inflammatory mediators upregulate tissue factor, a major initiator of the extrinsic coagulation pathway (7, 8). Aberrant tissue factor expression has been reported in eutopic and ectopic endometrium from women with endometriosis, consistent with local activation of coagulation-related pathways (9, 10). This microenvironment may favor thrombin generation, fibrin formation, and impaired fibrin clearance, thereby linking coagulation activity to fibrin deposition, fibrotic remodeling, and lesion persistence (11, 12).

Fibrin formation is a key downstream event in this pathway. Fibrinogen (FIB), the circulating precursor of fibrin, differs from global clotting-time measures such as activated partial thromboplastin time (APTT), prothrombin time (PT), and thrombin time (TT). As an acute-phase reactant, FIB may also reflect systemic inflammatory status, supporting its evaluation separately from clotting-time indices in endometriosis (12).

As a structural component of fibrinogen, fibrinogen alpha chain contributes to fibrin formation and clot architecture (12). In women with endometriosis, serum fibrinogen alpha chain levels were higher than those in controls, and increased expression was also observed in eutopic and ectopic endometrial tissues (13). Functional experiments further linked fibrinogen alpha chain to stromal-cell migration, invasion, matrix remodeling, and angiogenic signaling in endometriosis (13–15). Circulating plasma FIB may not directly mirror these tissue-level changes; it is also influenced by systemic inflammation and the hepatic acute-phase response (12). Whether tissue- and cell-level findings translate into plasma FIB levels in clinical cohorts remains uncertain.

Clinical studies have investigated coagulation abnormalities in endometriosis by comparing routine coagulation profiles between patients and controls. Comparative studies evaluated FIB together with clotting-time indices such as APTT, PT, and TT, and found changes compatible with a hypercoagulable phenotype in some patients (16–20). Other reports focused on deep infiltrating, ovarian, or advanced-stage endometriosis and assessed whether coagulation markers varied with disease phenotype or severity (21–23). Recent meta-analytic evidence further supports altered coagulation profiles in endometriosis (24). Control populations, covariate adjustment, and reporting of adjusted effect estimates varied across studies (16–23). Such heterogeneity limits direct application of these findings to women with infertility undergoing surgical evaluation, who may differ from healthy controls in reproductive, endocrine, and pelvic inflammatory backgrounds. FIB was often considered within a broader coagulation panel and was not evaluated as the primary exposure. It remains unclear whether plasma FIB is associated with surgically confirmed endometriosis, disease stage, or both in women with infertility.

The present study analyzed plasma FIB in women with infertility who underwent combined hysteroscopy and laparoscopy, with surgically confirmed endometriosis and disease stage as the main clinical outcomes. FIB was treated as the primary circulating coagulation indicator; APTT, PT, TT, and international normalized ratio (INR) were included as exploratory comparators. The aim was to determine whether plasma FIB is associated with the presence and stage of endometriosis in this surgically evaluated cohort of women with infertility.

## 2 Methods

### 2.1 Study design, setting, and participants

This retrospective cross-sectional study investigated the association between plasma fibrinogen (FIB) and surgically confirmed endometriosis among women with infertility undergoing combined hysteroscopy and laparoscopy. Other routine coagulation parameters were evaluated as exploratory comparators. The study was conducted at Foshan Fosun Chancheng Hospital, Foshan, Guangdong Province, China.

Eligible participants were consecutive women with infertility who underwent combined hysteroscopy and laparoscopy in the Department of TCM Gynecology, Foshan Fosun Chancheng Hospital, from January 1, 2019, to December 30, 2023. Ethical approval was obtained from the Ethics Committee of Foshan Fosun Chancheng Hospital (Approval No. CYEC-LCYJ-2024106-PJ-202401011). The requirement for informed consent was waived due to the retrospective design and the use of de-identified data.

**Figure 1:**
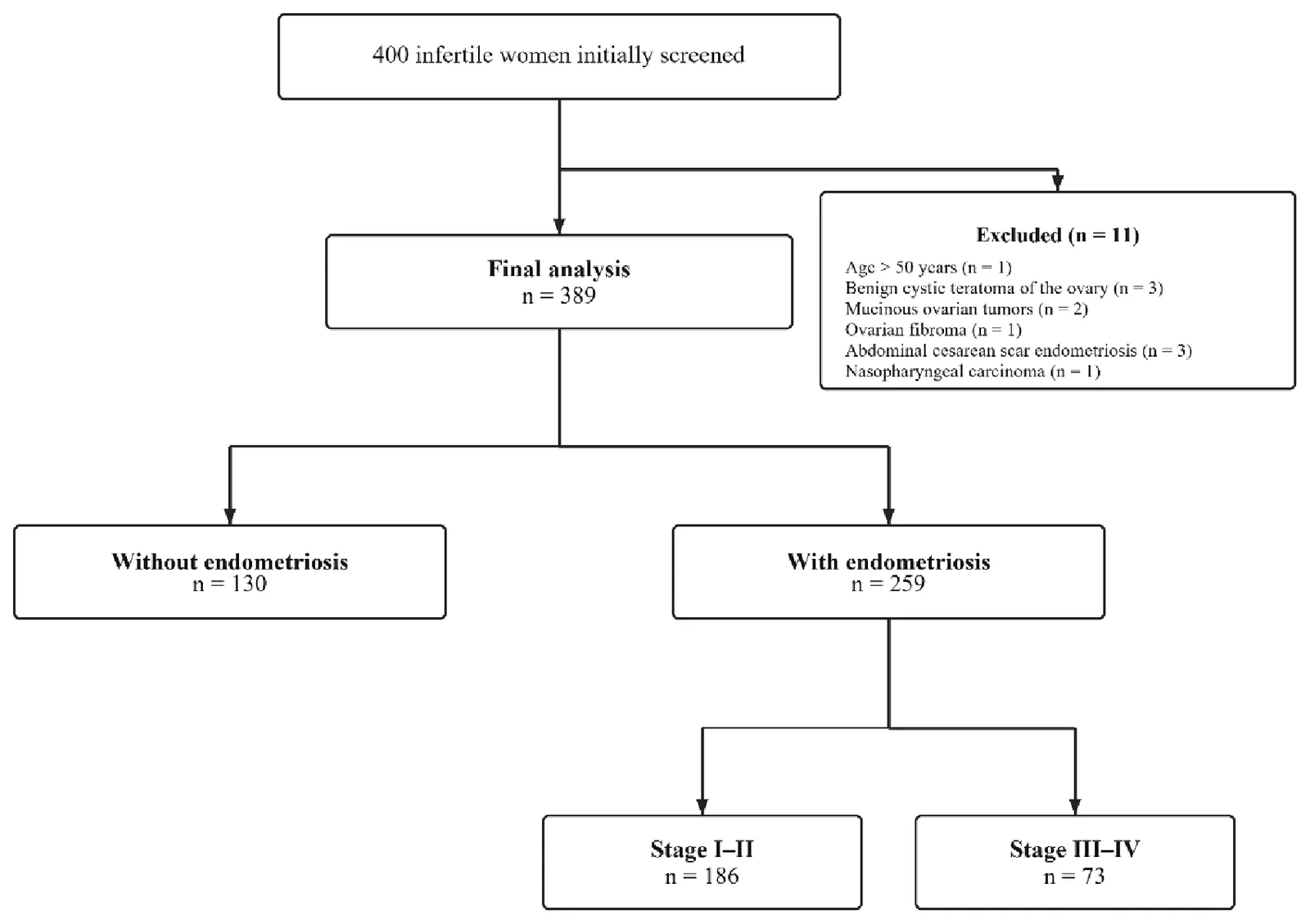
Flowchart of participant selection and classification. The flowchart shows participant screening, exclusion, final cohort inclusion, and classification according to endometriosis status and disease stage.

A total of 400 women were screened. Eleven women were excluded for the following reasons: age older than 50 years (n = 1), benign cystic teratoma of the ovary (n = 3), mucinous ovarian tumor (n = 2), ovarian fibroma (n = 1), abdominal cesarean scar endometriosis (n = 3), and nasopharyngeal carcinoma (n = 1). The final analytical cohort comprised 389 women: 130 without endometriosis and 259 with endometriosis, including 186 with stage I–II disease and 73 with stage III–IV disease (**Figure 1**).

### 2.2 Data collection

Sociodemographic and clinical information was extracted from the hospital electronic medical record system. Age, height, weight, education level, medical history, dysmenorrhea, gravidity, and parity were recorded at admission. Body mass index (BMI) was calculated as weight in kilograms divided by height in meters squared. Education level was categorized as junior high school or below, high school or technical school education, and college degree or above. Parity was categorized as nulliparous or multiparous. Smoking and alcohol consumption were recorded by self-report; all participants reported no smoking or alcohol consumption.

Additional reproductive and laboratory variables were extracted for sensitivity analyses. Menstrual-cycle phase at blood sampling was derived from the recorded last menstrual period and blood sampling date. Tubal factor status was derived from preoperative tubal patency records. PCOS and adenomyosis were identified from recorded clinical diagnoses. Preoperative white blood cell, neutrophil, lymphocyte, platelet, CA125, and D-dimer values were obtained from the medical records. The neutrophil-to-lymphocyte ratio (NLR) was calculated as the neutrophil count divided by the lymphocyte count, and the platelet-to-lymphocyte ratio (PLR) as the platelet count divided by the lymphocyte count.

### 2.3 Measurement of plasma coagulation parameters

Plasma coagulation parameters were obtained from the hospital electronic medical record system. Venous blood samples were collected from the antecubital vein one day before surgery into tubes containing 3.2% sodium citrate at a blood-to-anticoagulant ratio of 9:1.

The primary coagulation exposure was plasma FIB. Activated partial thromboplastin time (APTT), prothrombin time (PT), thrombin time (TT), and international normalized ratio (INR) were assessed as exploratory comparator parameters. All coagulation parameters were measured using standard coagulation assays on an automated coagulation analyzer (STA-R MAX, Stago, France) with corresponding reagents. The reference ranges were as follows: PT, 11.00–14.50 s; APTT, 28.00–43.50 s; TT, 14.00–21.00 s; FIB, 2.00–4.00 g/L; and INR, 0.80–1.30.

### 2.4 Diagnosis and staging of endometriosis

The primary clinical outcome was surgically confirmed endometriosis. Endometriosis status was determined from operative records and, where available, histopathological findings in the hospital electronic medical record system. The diagnosis of endometriosis was established according to laparoscopic and/or histopathological criteria (4, 5).

Endometriosis stage was classified using the revised American Society for Reproductive Medicine (rASRM) scoring system, which considers the size and depth of peritoneal and ovarian lesions, the extent of ovarian and fallopian tube adhesions, and the degree of posterior cul-de-sac obliteration (6). Based on the rASRM score, endometriosis was staged as stage I (minimal, 1–5 points), stage II (mild, 6–15 points), stage III (moderate, 16–40 points), and stage IV (severe, >40 points). For stage-specific analyses, stage I–II disease and stage III–IV disease were evaluated separately, with women without endometriosis used as the reference group.

### 2.5 Statistical analysis

Categorical variables were summarized as frequencies and percentages, and continuous variables as means and standard deviations or medians and interquartile ranges, as appropriate. For two-group comparisons, Student’s t test or the Mann–Whitney U test was used for continuous variables, and Pearson’s chi-square test or Fisher’s exact test for categorical variables. For comparisons across the three disease-status groups, one-way analysis of variance or the Kruskal–Wallis test was used for continuous variables, and Pearson’s chi-square test or an exact test for categorical variables.

Associations between plasma FIB and endometriosis were evaluated using binary logistic regression models. FIB was modeled as a continuous variable and as a categorical variable based on quartiles. Quartile cutoffs were derived from the distribution of FIB in the total study population, and the lowest quartile was used as the reference group. The primary model was adjusted for age, BMI, education level, dysmenorrhea, and parity. Covariates were selected *a priori* based on clinical relevance and data availability. Adjusted odds ratios (ORs) and 95% confidence intervals (CIs) were reported. P values for trend were calculated by assigning each participant the median FIB value of the corresponding quartile and modeling this value as a continuous variable.

Stage-specific associations were evaluated using separate logistic regression models comparing stage I–II endometriosis with no endometriosis and stage III–IV endometriosis with no endometriosis; cases from the other stage category were excluded from the corresponding model. The same covariate-adjustment strategy was used in these models. The distribution of no endometriosis, stage I–II endometriosis, and stage III–IV endometriosis across FIB quartiles was summarized descriptively.

Potential nonlinear associations between plasma FIB and endometriosis were examined using adjusted logistic regression models with penalized splines. The models were adjusted for the same covariates as the fully adjusted model. Similar exploratory spline analyses were performed for APTT, PT, TT, and INR as comparator coagulation parameters. Results for FIB were presented in the main text, whereas results for APTT, PT, TT, and INR were presented as supplementary analyses.

Sensitivity analyses were performed by adding selected reproductive, inflammatory, and coagulation-related variables to the primary model. Additional models separately adjusted for menstrual-cycle status; tubal factor status, PCOS, and adenomyosis; NLR; CA125; and D-dimer. Menstrual-cycle status was categorized as follicular, non-follicular, or unknown according to the derived cycle day at blood sampling. An exploratory expanded model additionally included NLR, PLR, CA125, and D-dimer together with the covariates in the primary model. This model was considered exploratory given the reduced sample size and potential model instability. Sensitivity analyses were based on available complete cases for each model.

Exploratory subgroup analyses were performed to assess the association between FIB quartiles and endometriosis across strata of age, BMI, education level, dysmenorrhea, and parity. Interactions between FIB quartiles and subgroup variables were assessed using likelihood ratio tests.

All data management and statistical analyses were performed using R software (version 4.4.0; R Foundation for Statistical Computing, Vienna, Austria) and EmpowerStats software (version 6.0; X&Y Solutions, Inc., Boston, MA, USA). All statistical tests were two-sided, and P < 0.05 was considered statistically significant.

## 3 Results

### 3.1 Baseline characteristics

Baseline characteristics are summarized in **Table 1**. Age and BMI were similar between women with and without endometriosis. The mean age was 31.95 ± 6.01 years in women without endometriosis and 31.94 ± 4.83 years in women with endometriosis (P = 0.575). The corresponding mean BMI values were 21.86 ± 3.28 and 21.23 ± 2.76 kg/m², respectively (P = 0.118). Education level differed between the two groups (P < 0.001), with a higher proportion of women with endometriosis having a college degree or above than women without endometriosis (68.34% vs. 48.46%). Dysmenorrhea was more frequent among women with endometriosis than among those without endometriosis (28.19% vs. 18.46%; P = 0.037). Parity did not differ significantly between the two groups (P = 0.618).

**Table 1:** Baseline characteristics of participants with and without endometriosis.

Characteristic	Without endometriosis (n = 130)	Endometriosis (n = 259)	P value
Age, years, mean ± SD	31.95 ± 6.01	31.94 ± 4.83	0.575
BMI, kg/m², mean ± SD	21.86 ± 3.28	21.23 ± 2.76	0.118
Education level, n (%)			<0.001
Junior high school or below	35 (26.92%)	26 (10.04%)	
High school or technical school education	32 (24.62%)	56 (21.62%)	
College degree or above	63 (48.46%)	177 (68.34%)	
Dysmenorrhea, n (%)			0.037
Yes	24 (18.46%)	73 (28.19%)	
No	106 (81.54%)	186 (71.81%)	
Parity, n (%)			0.618
Nulliparous	82 (63.08%)	170 (65.64%)	
Multiparous	48 (36.92%)	89 (34.36%)	

Additional clinical, reproductive, inflammatory, coagulation-related, and endometriosis-phenotype characteristics according to endometriosis status and disease stage are summarized in **Supplementary Table 1**. Menstrual-cycle status was similar across groups, with most blood samples obtained during the follicular phase. WBC, NLR, PLR, and D-dimer did not differ substantially across groups. CA125 levels, rASRM scores, and the proportion of ovarian endometrioma/chocolate cyst were higher in stage III–IV disease. Tubal factor status, PCOS, adenomyosis, and recorded comorbidities also varied across groups, and selected reproductive and laboratory variables were further considered in sensitivity analyses.

### 3.2 Association of plasma FIB with endometriosis and disease stage

The association of plasma FIB with endometriosis was evaluated using multivariable logistic regression models, with FIB analyzed as both a continuous variable and a quartile-based categorical variable (**Table 2**). In the fully adjusted model, each 1 g/L increase in FIB was not significantly associated with overall endometriosis (OR 1.20, 95% CI 0.76–1.91; P = 0.433).

**Table 2:** Adjusted associations of fibrinogen with overall and stage-specific endometriosis.

Outcome	FIB category	OR (95% CI)	P value	P for trend
Overall endometriosis	Continuous FIB, per 1 g/L	1.20 (0.76–1.91)	0.433	
	Q1: 0.86–2.40 g/L	Reference		0.178
	Q2: 2.41–2.68 g/L	1.27 (0.68–2.35)	0.449	
	Q3: 2.69–3.04 g/L	2.37 (1.20–4.66)	0.012	
	Q4: 3.06–4.63 g/L	1.48 (0.76–2.88)	0.244	
Stage I–II endometriosis	Continuous FIB, per 1 g/L	1.14 (0.71–1.84)	0.585	
	Q1: 0.86–2.40 g/L	Reference		0.290
	Q2: 2.41–2.68 g/L	1.34 (0.70–2.56)	0.383	
	Q3: 2.69–3.04 g/L	1.98 (0.97–4.03)	0.061	
	Q4: 3.06–4.63 g/L	1.42 (0.70–2.87)	0.329	
Stage III–IV endometriosis	Continuous FIB, per 1 g/L	1.56 (0.77–3.17)	0.221	
	Q1: 0.86–2.40 g/L	Reference		0.070
	Q2: 2.41–2.68 g/L	1.31 (0.51–3.38)	0.574	
	Q3: 2.69–3.04 g/L	4.03 (1.56–10.43)	0.004	
	Q4: 3.06–4.63 g/L	2.12 (0.77–5.80)	0.145	

Values are presented as odds ratios (95% confidence intervals). The fully adjusted model included age, BMI, education level, dysmenorrhea, and parity. FIB quartiles were defined according to the distribution of FIB in the overall analytical cohort and were applied consistently to overall and stage-specific analyses. P for trend was calculated by modeling the median FIB value of each quartile as a continuous variable. BMI, body mass index; CI, confidence interval; FIB, fibrinogen.

When FIB was categorized into quartiles, Q1 was used as the reference group. Compared with women in Q1 (0.86–2.40 g/L), those in Q2 (2.41–2.68 g/L) showed no significant difference in the odds of overall endometriosis (OR 1.27, 95% CI 0.68–2.35; P = 0.449). Women in Q3 (2.69–3.04 g/L) had higher odds of overall endometriosis than those in Q1 (OR 2.37, 95% CI 1.20–4.66; P = 0.012). The association for Q4 (3.06–4.63 g/L) was not statistically significant (OR 1.48, 95% CI 0.76–2.88; P = 0.244). The trend across FIB quartiles was also not significant (P for trend = 0.178).

**Figure 2:**
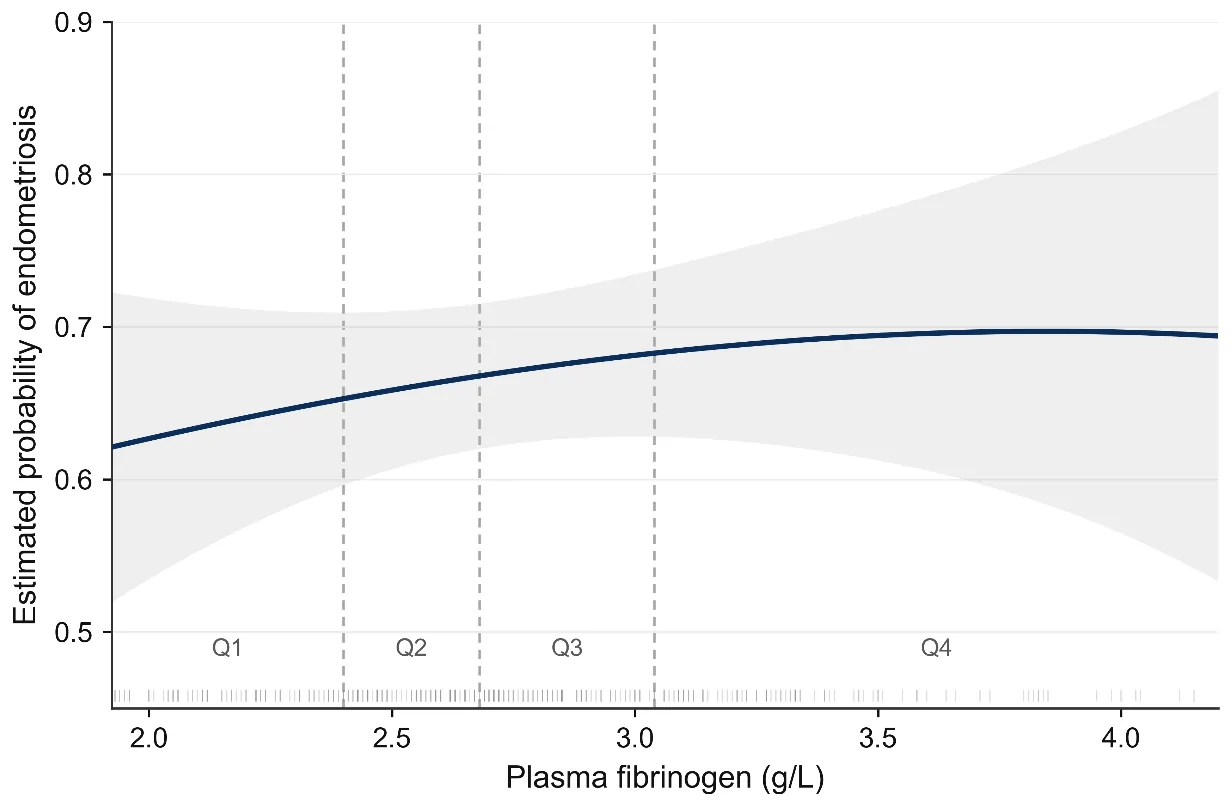
Adjusted spline curve for plasma fibrinogen and endometriosis. The solid line indicates the estimated probability of endometriosis, and the shaded area indicates the pointwise 95% confidence interval. Vertical dashed lines indicate fibrinogen quartile cutoffs.

The adjusted spline curve suggested an increase in the estimated probability of endometriosis across the lower-to-middle FIB range, followed by an apparent plateau at higher FIB levels (**Figure 2**). The pointwise 95% confidence intervals were wider at the lower and upper ends of the displayed FIB range, indicating greater uncertainty at the extremes. Together with the quartile-based estimates, this pattern did not support a simple monotonic dose–response relationship across the observed FIB distribution.

Stage-specific analyses showed different patterns for stage I–II and stage III–IV endometriosis (**Table 2**). For stage I–II disease, continuous FIB was not significantly associated with endometriosis status in the fully adjusted model (OR 1.14, 95% CI 0.71–1.84; P = 0.585). In the quartile model, Q3 showed a higher point estimate than Q1, although the association did not reach statistical significance (OR 1.98, 95% CI 0.97–4.03; P = 0.061). Q2 and Q4 were also not significant, and the trend across quartiles was not significant (P for trend = 0.290).

For stage III–IV disease, continuous FIB was not significantly associated with endometriosis status (OR 1.56, 95% CI 0.77–3.17; P = 0.221). In contrast, women in Q3 had higher odds of stage III–IV endometriosis than those in Q1 (OR 4.03, 95% CI 1.56–10.43; P = 0.004). The associations for Q2 and Q4 were not statistically significant, with ORs of 1.31 (95% CI 0.51–3.38; P = 0.574) and 2.12 (95% CI 0.77–5.80; P = 0.145), respectively. The trend across FIB quartiles did not reach statistical significance (P for trend = 0.070).

**Figure 3:**
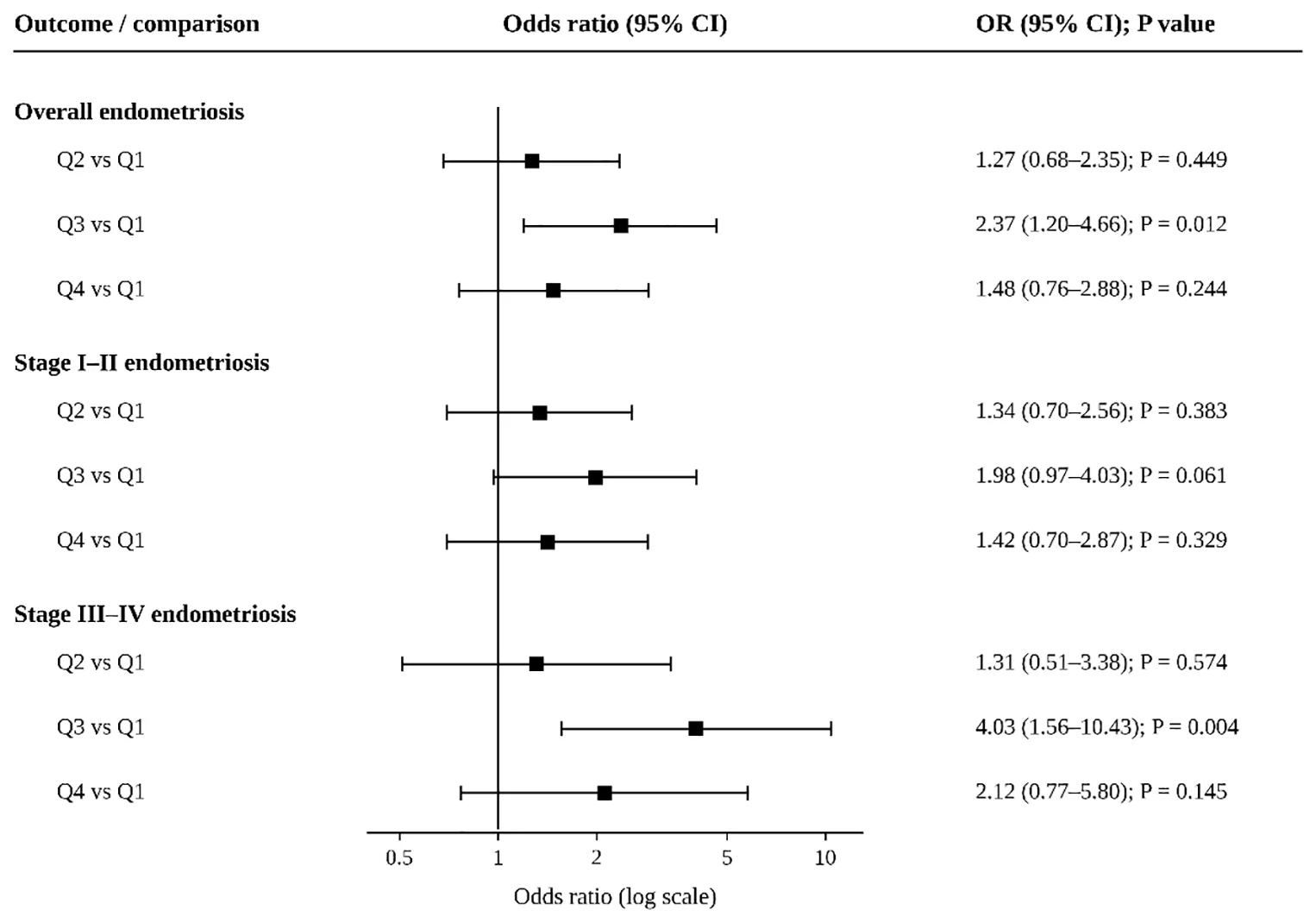
Adjusted odds ratios for overall and stage-specific endometriosis according to fibrinogen quartiles. Estimates were obtained from the fully adjusted model, with Q1 as the reference group. Error bars indicate 95% confidence intervals. The x-axis is shown on a logarithmic scale.

The forest plot provided a visual summary of the fully adjusted quartile-based estimates (**Figure 3**). Across overall endometriosis and both stage-specific outcomes, the highest point estimate was observed in Q3, most notably for stage III–IV disease, whereas the confidence intervals for Q2 and Q4 generally crossed the null value.

**Figure 4:**
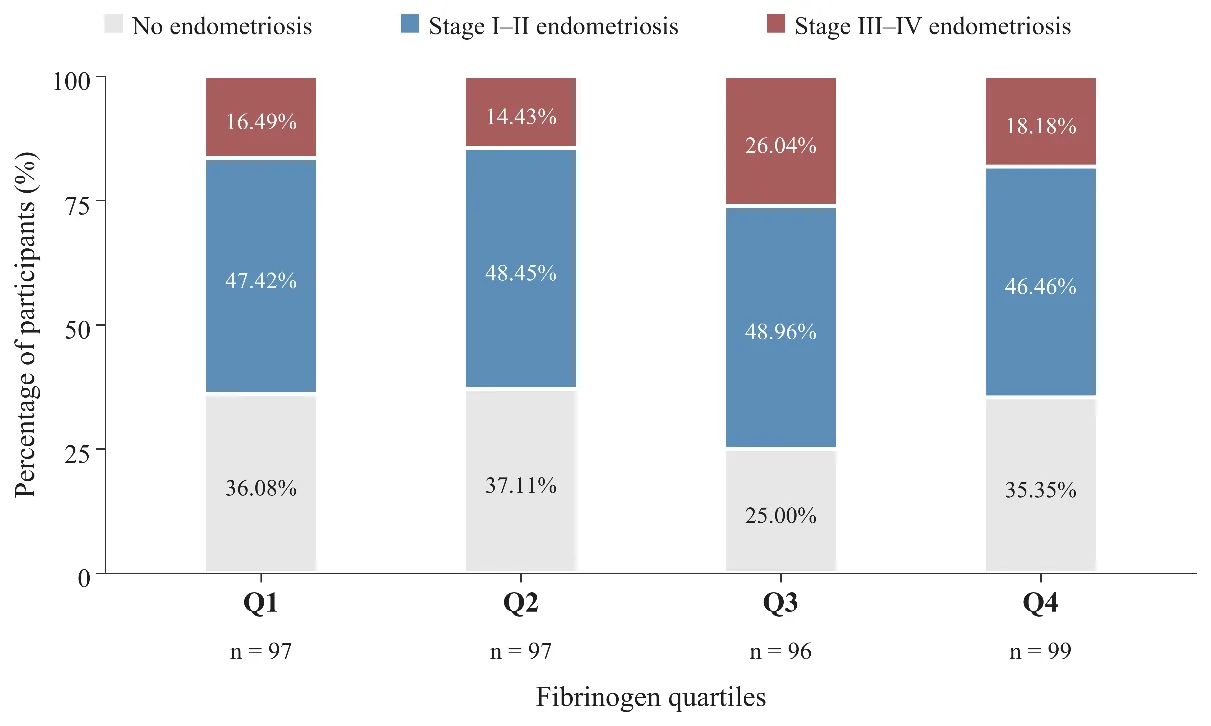
Distribution of endometriosis status and disease stage across fibrinogen quartiles. Stacked bars show the proportions of women without endometriosis, with stage I–II endometriosis, and with stage III–IV endometriosis within each fibrinogen quartile.

The distribution of endometriosis status and disease stage across FIB quartiles further illustrated this pattern (**Figure 4**). The proportion of stage III–IV endometriosis was highest in Q3, accounting for 26.0% of participants in that quartile, compared with 16.5% in Q1, 14.4% in Q2, and 18.2% in Q4. The proportion of stage I–II endometriosis was relatively stable across quartiles. These descriptive findings did not suggest a simple stepwise increase in disease stage across increasing FIB quartiles.

Sensitivity analyses are summarized in **Supplementary Table 2**. Additional adjustment for menstrual-cycle status; tubal factor status, PCOS, and adenomyosis; NLR; CA125; or D-dimer produced broadly similar overall quartile estimates. In the exploratory expanded model, continuous FIB remained unassociated with overall endometriosis (OR 0.98, 95% CI 0.58–1.65), whereas the Q3 estimate remained elevated (OR 2.27, 95% CI 1.09–4.75), with no significant trend across quartiles (P for trend = 0.533). For stage III–IV disease, the Q3 estimate was attenuated in the expanded model (OR 2.84, 95% CI 0.96–8.35).

### 3.3 Exploratory s of other coagulation parameters and subgroup s

APTT, PT, TT, and INR were evaluated as comparator coagulation parameters. In the fully adjusted models, no parameter was significantly associated with endometriosis when analyzed as a continuous variable: APTT (OR, 1.01; 95% CI, 0.94–1.08; P = 0.780), PT (OR, 1.06; 95% CI, 0.76–1.46; P = 0.740), TT (OR, 0.99; 95% CI, 0.87–1.11; P = 0.832), and INR (OR, 0.56; 95% CI, 0.03–10.47; P = 0.700). Quartile-based analyses showed no consistent associations with endometriosis, and spline analyses did not indicate clear nonlinear patterns. Detailed results are provided in **Supplementary Table 3** and **Supplementary Figure 1**.

Subgroup analyses were performed to evaluate whether the association between fibrinogen quartiles and endometriosis varied by age, BMI, education level, dysmenorrhea, or parity. No statistically significant interactions were observed for age, education level, dysmenorrhea, or parity. The interaction for BMI could not be estimated due to sparse data in the higher-BMI subgroup. The subgroup results are summarized in **Supplementary Table 4**.

## 4 Discussion

In this retrospective cross-sectional study of women with infertility undergoing combined hysteroscopy and laparoscopy, the association between plasma FIB and endometriosis differed across modeling approaches. Continuous FIB was not significantly associated with overall or stage-specific endometriosis. Quartile-based analyses, in contrast, showed higher estimates in Q3, particularly for stage III–IV disease. Q4 did not show a further increase, and the tests for trend across quartiles were not statistically significant. The adjusted spline increased across the lower-to-middle FIB range and then plateaued rather than showing a distinct Q3 peak. APTT, PT, TT, and INR showed no consistent associations. The overall findings suggest that the association between plasma FIB and endometriosis is not well described by a simple linear dose–response model. An elevated estimate in the intermediate FIB range persisted across several analyses, particularly for stage III–IV disease. This observation remains exploratory and requires independent confirmation.

Previous studies have reported coagulation abnormalities in women with endometriosis, including findings compatible with a hypercoagulable phenotype. Several studies evaluated FIB together with clotting-time measures such as APTT, PT, and TT and reported altered coagulation profiles in some patients with endometriosis (16–20). Other reports focused on deep infiltrating, ovarian, or advanced-stage disease and examined whether coagulation markers differed by phenotype or severity (21–23). A recent meta-analysis also supported abnormal coagulation profiles in endometriosis (24). Differences in control selection, clinical phenotype, sample size, covariate adjustment, and reporting limit direct comparison across studies. Most previous studies compared routine coagulation profiles between patients and controls or across selected disease phenotypes. In our cohort, both women with and without endometriosis underwent surgical evaluation, reducing the likelihood of outcome misclassification from an unverified reference group. FIB was examined using continuous, quartile-based, spline-based, and stage-specific analyses, with conventional clotting-time measures evaluated separately.

A biological link between FIB and the inflammatory–coagulation processes involved in endometriosis is plausible. Endometriosis is characterized by chronic inflammation, cyclic bleeding, tissue repair, and fibrotic remodeling. Inflammatory mediators can upregulate tissue factor, activate the extrinsic coagulation pathway, and promote thrombin generation (7–10). Fibrin formation is a downstream process implicated in fibrin deposition, adhesion formation, extracellular matrix remodeling, and lesion persistence (11, 12). FIB is both the circulating precursor of fibrin and an acute-phase reactant. Experimental studies have reported increased fibrinogen alpha-chain expression in serum, eutopic endometrium, and ectopic endometrial tissues from women with endometriosis (13–15). Functional studies have linked fibrinogen alpha chain to stromal-cell migration, invasion, matrix remodeling, and angiogenic signaling. Plasma FIB should not be regarded as a surrogate for tissue fibrinogen alpha-chain expression or lesion-level inflammatory activity. In the present cohort, WBC, NLR, PLR, and D-dimer did not differ substantially across disease-stage groups. CA125 was higher in stage III–IV disease. Additional adjustment for NLR, CA125, or D-dimer produced little change in the overall Q3 estimate. These findings make it less likely that the Q3 association was explained entirely by these measured variables. They do not establish an inflammation-mediated mechanism. More specific inflammatory measures, including CRP and cytokine profiles, were unavailable.

The largest estimate was observed in Q3 (2.69–3.04 g/L), which lies entirely within the laboratory reference range of 2.00–4.00 g/L. The quartile boundaries were distribution-based rather than prespecified clinical thresholds, and the spline showed an increase followed by a plateau rather than a discrete Q3 peak. Multiple quartile comparisons across overall and stage-specific outcomes also add uncertainty to the isolated Q3 associations. The Q3 finding is best interpreted as a distribution-dependent observation rather than a biological or clinical cutoff. The intermediate-range pattern was also not confined to the primary model. Adjustment for menstrual-cycle status produced estimates similar to those of the primary analysis, and the overall Q3 estimate remained elevated after additional adjustment for tubal factor status, PCOS, and adenomyosis and after separate adjustment for NLR, CA125, and D-dimer. In the exploratory expanded model, the Q3 estimate for overall endometriosis remained elevated (OR 2.27, 95% CI 1.09–4.75). Continuous FIB remained null (OR 0.98, 95% CI 0.58–1.65), and the trend across quartiles was not significant (P for trend = 0.533). These sensitivity analyses show that the intermediate-range pattern was reasonably consistent across model specifications, although a graded exposure–response relationship was not observed.

Stage-specific estimates were less precise. The primary Q3 estimate for stage III–IV disease was OR 4.03 (95% CI 1.56–10.43), based on only 73 stage III–IV cases. In the exploratory expanded model, the estimate decreased to 2.84 (95% CI 0.96–8.35) and no longer reached conventional statistical significance. The stage III–IV finding was less precise and more sensitive to model specification than the overall Q3 result. Advanced endometriosis is characterized by more extensive lesions, adhesions, fibrotic remodeling, and pelvic anatomical distortion. These features provide a biological context for examining fibrinogen-related processes across disease phenotypes. rASRM stage primarily reflects lesion distribution, lesion size, adhesions, and cul-de-sac obliteration and does not fully capture lesion activity, inflammatory intensity, pain severity, or mechanisms of infertility. The larger Q3 estimate in stage III–IV disease provides a rationale for further evaluation of FIB across anatomical disease phenotypes, without supporting its use as an individual staging marker.

Several reproductive and clinical factors can influence circulating FIB. The median cycle day was day 9 in all three disease categories, and more than 90% of samples in each group were obtained during the follicular phase. Menstrual-cycle phase did not differ significantly across groups, and adjustment for menstrual-cycle status had little effect on the estimates. Tubal factor status, PCOS, and adenomyosis differed across disease groups, and adjustment for these factors did not materially alter the overall Q3 estimate. Recorded hypertension and diabetes were uncommon. Information on preoperative hormonal therapy was not systematically recorded in the retrospective dataset and could not be reliably classified or included in the adjusted analyses. Hormonal exposure can influence coagulation parameters, leaving open the possibility of residual confounding related to treatment. The direction or magnitude of any resulting bias cannot be determined from the available data.

APTT, PT, TT, and INR were not consistently associated with endometriosis, and the quartile-specific pattern observed for FIB was not reproduced by these conventional clotting-time measures. APTT, PT, and TT are global coagulation tests designed primarily to identify clinically relevant abnormalities in coagulation pathways. FIB differs from these measures as a concentration-based plasma protein and an acute-phase reactant. INR requires separate interpretation, as it is derived from PT and does not represent an independent coagulation pathway measure. Its analysis was exploratory. The observed FIB pattern was not mirrored by the routine clotting-time measures examined in this cohort. This difference supports evaluating FIB separately from conventional clotting-time indices in future studies, without implying a distinct biological pathway. Plasma FIB cannot yet be considered a standalone diagnostic or staging marker. Future work should determine whether the observed pattern is reproducible and whether FIB adds information beyond standard coagulation tests, clinical features, imaging findings, CA125, or other inflammatory, coagulation, and fibrinolytic markers.

The study has several strengths. Endometriosis status and stage were surgically assessed in all participants, reducing the likelihood of undiagnosed endometriosis in the control group. FIB was defined as the primary coagulation exposure, while APTT, PT, TT, and INR were analyzed as comparator parameters. Continuous, quartile-based, spline-based, stage-specific, and subgroup analyses were used to characterize the association pattern, with additional sensitivity analyses for available reproductive and laboratory variables.

Several limitations remain. The retrospective cross-sectional design precludes causal inference and cannot determine whether FIB changes preceded or followed endometriosis development. The single-center setting and restriction to women with infertility undergoing surgery limit the generalizability of the findings. Selection and ascertainment bias are also possible in this retrospective surgical cohort. The marked difference in education level could partly reflect referral or selection processes. Education was included in the adjusted model; residual selection bias cannot be excluded. Information on dysmenorrhea was retrospectively extracted from routine admission records and was documented in 28.2% of women with endometriosis. Symptom assessment was based on routine clinical documentation rather than a standardized questionnaire; under-recording cannot be excluded, and the observed proportion should be interpreted as the documented frequency rather than the true prevalence of dysmenorrhea in this cohort. Detailed infertility etiology, including male-factor infertility and unexplained infertility, was not systematically captured in the dataset, although tubal factor status and PCOS were available for sensitivity analyses. Residual confounding from incompletely measured clinical factors also remains possible. Menstrual-cycle timing, tubal factor status, PCOS, adenomyosis, NLR, CA125, and D-dimer were examined in sensitivity analyses, but information on hormonal therapy, anti-inflammatory medication use, acute infection, liver function, metabolic factors, CRP, and detailed treatment history was unavailable or insufficient for reliable adjustment. FIB was measured only once before surgery, and dynamic changes were not assessed. More specific mechanistic markers, including tissue factor, thrombin-generation measures, plasminogen activator system components, inflammatory cytokines, tissue-level fibrin deposition, and fibrinogen alpha-chain expression, were unavailable. The number of stage III–IV cases was limited. The extent of missingness differed across supplementary variables, and sensitivity analyses were based on complete cases for each model. Complete-case analysis may introduce selection bias if data availability is related to participant characteristics or disease status. Sample size decreased from 389 in the primary overall model to 353 in the exploratory expanded model and from 203 to 181 in the corresponding stage III–IV models, with reduced precision, particularly in the stage-specific analyses. Quartile-based, stage-specific, and subgroup analyses were exploratory and were not adjusted for multiple comparisons.

Prospective studies with standardized blood-sampling schedules, detailed hormonal-treatment records, repeated FIB measurements, and more specific inflammatory and coagulation markers are needed to determine whether the intermediate-range FIB pattern is reproducible. Parallel assessment of plasma FIB, tissue fibrinogen alpha-chain expression, fibrin deposition, tissue factor, thrombin-generation markers, and fibrinolytic pathways could clarify the relationship between circulating FIB and lesion biology. Such studies could determine whether the observed intermediate-range pattern is reproducible across populations or reflects cohort-specific variation, and whether FIB provides information beyond routinely available clinical and laboratory measures.

## 5 Conclusion

In women with infertility undergoing surgical evaluation, plasma FIB did not show a simple linear association with endometriosis. A higher estimate in the intermediate FIB range was observed across quartile-based analyses and persisted across several adjusted models, with the largest estimate in stage III–IV disease. Trends across quartiles were not significant, and APTT, PT, TT, and INR showed no consistent associations. This intermediate-range pattern remains exploratory and requires confirmation in independent cohorts before its clinical relevance can be established.

## Data Availability

The raw data supporting the conclusions of this article will be made available by the authors, without undue reservation.
