# Supplementary material for: Plasma fibrinogen levels in women with infertility undergoing surgical evaluation for endometriosis: a retrospective cross-sectional study
Source: Front Endocrinol (Lausanne). 2026 Sep 10;17:1929933. doi: 10.3389/fendo.2026.1929933 (PMC13600913; doi:10.3389/fendo.2026.1929933)
Supplement: Supplementary file 1 [file Supplementaryfile1.docx]

**Supplementary Materials**

**Supplementary tables**

**Table S1. Additional clinical, reproductive, inflammatory, coagulation-related, and disease-phenotype characteristics according to endometriosis status and disease stage**

| **Characteristic** | **No endometriosis (n = 130)** | **Stage I-II (n = 186)** | **Stage III-IV (n = 73)** | **P value** |
| --- | --- | --- | --- | --- |
| Age, years, mean ± SD | 31.95 ± 6.01 | 32.04 ± 4.92 | 31.68 ± 4.63 | 0.888 |
| BMI, kg/m², mean ± SD | 21.86 ± 3.28 | 21.35 ± 2.87 | 20.91 ± 2.45 | 0.077 |
| Infertility duration, years, median (IQR) | 2.00 (1.00, 4.00) | 2.00 (1.00, 3.00) | 1.00 (1.00, 3.00) | 0.070 |
| Cycle day at blood sampling, median (IQR) | 9.00 (8.00, 10.00) | 9.00 (8.00, 10.00) | 9.00 (8.00, 10.00) | 0.845 |
| Menstrual-cycle phase, n (%) |  |  |  | 0.586 |
| Menstrual phase, days 1-5 | 4 (3.1%) | 4 (2.2%) | 2 (2.7%) |  |
| Follicular phase, days 6-13 | 121 (93.1%) | 171 (91.9%) | 68 (93.2%) |  |
| Ovulatory window, days 14-16 | 0 (0.0%) | 0 (0.0%) | 1 (1.4%) |  |
| Luteal phase, days 17-35 | 1 (0.8%) | 1 (0.5%) | 0 (0.0%) |  |
| Unknown/not recorded | 4 (3.1%) | 10 (5.4%) | 2 (2.7%) |  |
| Primary infertility, n (%) | 58 (44.6%) | 91 (48.9%) | 40 (54.8%) | 0.376 |
| Tubal factor status, n (%) |  |  |  | <0.001 |
| Yes | 75 (57.7%) | 54 (29.0%) | 11 (15.1%) |  |
| No | 40 (30.8%) | 125 (67.2%) | 52 (71.2%) |  |
| Unknown/not recorded | 15 (11.5%) | 7 (3.8%) | 10 (13.7%) |  |
| PCOS, n (%) | 18 (13.8%) | 18 (9.7%) | 1 (1.4%) | 0.015 |
| Adenomyosis, n (%) | 17 (13.1%) | 39 (21.0%) | 36 (49.3%) | <0.001 |
| Any recorded comorbidity, n (%) | 20 (15.4%) | 24 (12.9%) | 2 (2.7%) | 0.023 |
| Hypertension, n (%) | 1 (0.8%) | 1 (0.5%) | 0 (0.0%) | — |
| Diabetes, n (%) | 0 (0.0%) | 1 (0.5%) | 0 (0.0%) | — |
| Other recorded comorbidity, n (%) | 1 (0.8%) | 4 (2.2%) | 1 (1.4%) | — |
| WBC, ×10⁹/L, median (IQR) | 6.30 (5.20, 7.30) | 6.30 (5.30, 7.38) | 6.00 (5.30, 6.90) | 0.350 |
| Neutrophil count, ×10⁹/L, median (IQR) | 3.43 (2.67, 4.19) | 3.50 (2.80, 4.29) | 3.20 (2.70, 4.00) | 0.498 |
| Lymphocyte count, ×10⁹/L, median (IQR) | 2.16 (1.78, 2.55) | 2.20 (1.80, 2.56) | 2.10 (1.80, 2.50) | 0.722 |
| Platelet count, ×10⁹/L, median (IQR) | 278.00 (238.00, 313.00) | 262.00 (229.25, 295.75) | 274.00 (215.00, 306.00) | 0.081 |
| NLR, median (IQR) | 1.61 (1.20, 2.05) | 1.60 (1.29, 2.00) | 1.60 (1.29, 1.95) | 0.900 |
| PLR, median (IQR) | 126.80 (101.36, 162.94) | 120.89 (100.00, 144.42) | 127.08 (106.46, 152.26) | 0.168 |
| CA125, U/mL, median (IQR) | 12.54 (8.98, 17.77) | 13.96 (9.46, 21.54) | 35.24 (19.14, 62.79) | <0.001 |
| D-dimer, μg/mL, median (IQR) | 0.62 (0.41, 0.77) | 0.62 (0.43, 0.78) | 0.63 (0.42, 0.77) | 0.953 |
| rASRM score, median (IQR) | Not applicable | 2.00 (2.00, 3.00) | 30.00 (22.00, 50.00) | <0.001 |
| Ovarian endometrioma/chocolate cyst, n (%) | 0 (0.0%) | 2 (1.1%) | 72 (98.6%) | <0.001 |

Note. Values are presented as mean ± SD, median (IQR), or n (%), as appropriate. Menstrual-cycle phase was derived from the last menstrual period and blood sampling date; invalid or uninterpretable dates were coded as unknown/not recorded. Tubal factor status was derived from preoperative tubal patency records; cases without interpretable tubal patency information were coded as unknown/not recorded. Primary infertility was defined as no previous pregnancy recorded in the dataset. PCOS, hypertension, diabetes, and other comorbidities were based on recorded comorbidity fields. NLR was calculated as the neutrophil count divided by the lymphocyte count, and PLR as the platelet count divided by the lymphocyte count. P values were calculated using one-way ANOVA for normally distributed continuous variables, the Kruskal–Wallis test for non-normally distributed continuous variables, and chi-square or exact tests for categorical variables, as appropriate. The P value for the rASRM score compares stage I–II and stage III–IV disease only. P values were not reported for hypertension, diabetes, and other recorded comorbidities due to sparse events. Abbreviations: BMI, body mass index; CA125, cancer antigen 125; IQR, interquartile range; NLR, neutrophil-to-lymphocyte ratio; PCOS, polycystic ovary syndrome; PLR, platelet-to-lymphocyte ratio; rASRM, revised American Society for Reproductive Medicine; SD, standard deviation; WBC, white blood cell count.

**Table S2. Sensitivity analyses for the association between plasma fibrinogen and endometriosis after additional adjustment for reproductive, inflammatory, and coagulation-related variables**

**Overall endometriosis**

| **Model** | **n** | **Continuous FIB** | **Q2 vs Q1** | **Q3 vs Q1** | **Q4 vs Q1** | **P for trend** |
| --- | --- | --- | --- | --- | --- | --- |
| Original model | 389 | 1.20 (0.76–1.91) | 1.27 (0.68–2.35) | 2.37 (1.20–4.66) | 1.48 (0.76–2.88) | 0.178 |
| + menstrual-cycle status | 389 | 1.20 (0.75–1.91) | 1.26 (0.68–2.34) | 2.35 (1.19–4.63) | 1.47 (0.76–2.86) | 0.186 |
| + tubal factor status, PCOS, and adenomyosis | 389 | 1.20 (0.73–1.96) | 1.04 (0.53–2.05) | 2.28 (1.10–4.72) | 1.27 (0.62–2.60) | 0.339 |
| + NLR | 388 | 1.17 (0.73–1.88) | 1.31 (0.71–2.44) | 2.36 (1.20–4.63) | 1.46 (0.75–2.85) | 0.202 |
| + CA125 | 382 | 1.12 (0.70–1.79) | 1.27 (0.68–2.37) | 2.27 (1.14–4.50) | 1.38 (0.71–2.70) | 0.262 |
| + D-dimer | 359 | 1.17 (0.72–1.93) | 1.25 (0.66–2.37) | 2.55 (1.25–5.18) | 1.42 (0.71–2.83) | 0.232 |
| Exploratory expanded model | 353 | 0.98 (0.58–1.65) | 1.10 (0.56–2.16) | 2.27 (1.09–4.75) | 1.15 (0.56–2.36) | 0.533 |

**Stage I–II endometriosis**

| **Model** | **n** | **Continuous FIB** | **Q2 vs Q1** | **Q3 vs Q1** | **Q4 vs Q1** | **P for trend** |
| --- | --- | --- | --- | --- | --- | --- |
| Original model | 316 | 1.14 (0.71–1.84) | 1.34 (0.70–2.56) | 1.98 (0.97–4.03) | 1.42 (0.70–2.87) | 0.290 |
| + menstrual-cycle status | 316 | 1.13 (0.70–1.82) | 1.31 (0.68–2.52) | 1.93 (0.94–3.96) | 1.39 (0.69–2.82) | 0.317 |
| + tubal factor status, PCOS, and adenomyosis | 316 | 1.18 (0.71–1.97) | 1.10 (0.54–2.23) | 2.08 (0.97–4.47) | 1.27 (0.60–2.70) | 0.396 |
| + NLR | 315 | 1.10 (0.68–1.79) | 1.40 (0.72–2.69) | 1.97 (0.97–4.02) | 1.38 (0.68–2.80) | 0.338 |
| + CA125 | 309 | 1.09 (0.68–1.77) | 1.36 (0.71–2.63) | 2.00 (0.97–4.12) | 1.38 (0.68–2.78) | 0.337 |
| + D-dimer | 292 | 1.06 (0.63–1.77) | 1.29 (0.66–2.54) | 2.04 (0.97–4.30) | 1.29 (0.61–2.69) | 0.429 |
| Exploratory expanded model | 286 | 0.97 (0.57–1.66) | 1.31 (0.64–2.66) | 1.98 (0.92–4.27) | 1.25 (0.58–2.67) | 0.495 |

**Stage III–IV endometriosis**

| **Model** | **n** | **Continuous FIB** | **Q2 vs Q1** | **Q3 vs Q1** | **Q4 vs Q1** | **P for trend** |
| --- | --- | --- | --- | --- | --- | --- |
| Original model | 203 | 1.56 (0.77–3.17) | 1.31 (0.51–3.38) | 4.03 (1.56–10.43) | 2.12 (0.77–5.80) | 0.070 |
| + menstrual-cycle status | 203 | 1.59 (0.78–3.25) | 1.29 (0.49–3.38) | 4.25 (1.61–11.24) | 2.19 (0.79–6.04) | 0.065 |
| + tubal factor status, PCOS, and adenomyosis | 203 | 1.36 (0.57–3.28) | 1.28 (0.39–4.23) | 3.70 (1.11–12.30) | 2.04 (0.60–6.93) | 0.181 |
| + NLR | 202 | 1.58 (0.77–3.27) | 1.34 (0.52–3.46) | 4.08 (1.57–10.58) | 2.16 (0.78–5.98) | 0.068 |
| + CA125 | 197 | 1.32 (0.63–2.76) | 1.17 (0.44–3.08) | 3.23 (1.23–8.44) | 1.64 (0.58–4.65) | 0.195 |
| + D-dimer | 186 | 1.66 (0.80–3.42) | 1.34 (0.48–3.68) | 4.84 (1.77–13.23) | 2.13 (0.75–6.06) | 0.072 |
| Exploratory expanded model | 181 | 1.07 (0.47–2.44) | 0.77 (0.24–2.43) | 2.84 (0.96–8.35) | 1.09 (0.34–3.47) | 0.585 |

**Note.** Values are presented as odds ratios (95% confidence intervals). The original model was adjusted for age, BMI, education level, dysmenorrhea, and parity. FIB quartiles were defined according to the distribution of FIB in the overall analytical cohort and were applied consistently to overall and stage-specific analyses. Additional models further adjusted for menstrual-cycle status, tubal factor status, PCOS, adenomyosis, NLR, CA125, or D-dimer, as indicated. Menstrual-cycle status was categorized as follicular, non-follicular, or unknown according to the derived cycle day at blood sampling. Tubal factor status was derived from preoperative tubal patency records. The exploratory expanded model additionally included NLR, PLR, CA125, and D-dimer and was considered exploratory given the reduced sample size and potential model instability. P for trend was calculated by modeling the median FIB value of each quartile as a continuous variable. Analyses were based on complete cases for each model. In the overall cohort, NLR was available for 388/389 participants (99.7%), PLR for 388/389 (99.7%), CA125 for 382/389 (98.2%), and D-dimer for 359/389 (92.3%). The exploratory expanded model included 353/389 participants (90.7%) with complete data for all variables included in the model.

**Abbreviations:** BMI, body mass index; CA125, cancer antigen 125; CI, confidence interval; FIB, fibrinogen; NLR, neutrophil-to-lymphocyte ratio; OR, odds ratio; PCOS, polycystic ovary syndrome; PLR, platelet-to-lymphocyte ratio.

**Table S3. Associations of APTT, PT, TT, and INR with endometriosis**

| **Measure** | **Category** | **n/cases** | **OR (95% CI)** | **P value** |
| --- | --- | --- | --- | --- |
| **APTT** | Continuous | 389/259 | 1.01 (0.94, 1.08) | 0.780 |
|  | Q1: 31.10–36.50 s | 94/59 | Reference |  |
|  | Q2: 36.60–38.50 s | 97/68 | 1.35 (0.71, 2.54) | 0.358 |
|  | Q3: 38.60–40.80 s | 100/71 | 1.31 (0.69, 2.47) | 0.409 |
|  | Q4: 40.90–54.00 s | 98/61 | 1.03 (0.55, 1.94) | 0.921 |
|  | P for trend |  |  | 0.997 |
| **PT** | Continuous | 389/259 | 1.06 (0.76, 1.46) | 0.740 |
|  | Q1: 11.20–12.60 s | 89/57 | Reference |  |
|  | Q2: 12.70–13.00 s | 101/65 | 1.00 (0.54, 1.87) | 0.993 |
|  | Q3: 13.10–13.50 s | 96/67 | 1.26 (0.66, 2.39) | 0.488 |
|  | Q4: 13.60–17.90 s | 103/70 | 1.07 (0.56, 2.02) | 0.839 |
|  | P for trend |  |  | 0.787 |
| **TT** | Continuous | 389/259 | 0.99 (0.87, 1.11) | 0.832 |
|  | Q1: 14.60–16.50 s | 85/55 | Reference |  |
|  | Q2: 16.60–17.10 s | 93/65 | 1.37 (0.71, 2.62) | 0.345 |
|  | Q3: 17.20–17.80 s | 111/80 | 1.34 (0.71, 2.51) | 0.368 |
|  | Q4: 17.90–45.90 s | 100/59 | 0.83 (0.44, 1.57) | 0.574 |
|  | P for trend |  |  | 0.523 |
| **INR** | Continuous | 389/259 | 0.56 (0.03, 10.47) | 0.700 |
|  | Q1: 0.80–0.94 | 93/59 | Reference |  |
|  | Q2: 0.95–0.98 | 99/67 | 1.25 (0.67, 2.33) | 0.482 |
|  | Q3: 0.99–1.03 | 90/67 | 1.71 (0.88, 3.33) | 0.113 |
|  | Q4: 1.04–1.47 | 107/66 | 0.87 (0.47, 1.59) | 0.642 |
|  | P for trend |  |  | 0.605 |

Abbreviations: APTT, activated partial thromboplastin time; CI, confidence interval; INR, international normalized ratio; OR, odds ratio; PT, prothrombin time; TT, thrombin time.

Note: Estimates were obtained from the fully adjusted model, which adjusted for age, BMI, education level, dysmenorrhea, and parity. The lowest quartile was used as the reference group. P for trend was calculated by modeling the median value of each quartile as a continuous variable. Continuous estimates are expressed per 1-second increase for APTT, PT, and TT and per 1-unit increase for INR.

**Table S4. Subgroup analyses for the association between fibrinogen quartiles and endometriosis**

| **Subgroup** | **n/cases** | **Q1** | **Q2 OR (95% CI)** | **Q3 OR (95% CI)** | **Q4 OR (95% CI)** | **P for interaction** |
| --- | --- | --- | --- | --- | --- | --- |
| Age <35 years | 277/193 | Reference | 1.33 (0.62, 2.86) | 2.01 (0.88, 4.63) | 1.16 (0.50, 2.67) | 0.299 |
| Age ≥35 years | 112/66 | Reference | 0.54 (0.15, 1.87) | 1.85 (0.50, 6.88) | 1.90 (0.57, 6.36) |  |
| BMI <25 kg/m² | 349/240 | Reference | 1.29 (0.69, 2.42) | 2.19 (1.09, 4.38) | 1.70 (0.85, 3.43) | NA |
| BMI ≥25 kg/m² | 40/19 | NA | Reference | 2.88 (0.24, 34.07) | 0.30 (0.03, 2.97) |  |
| Junior high school or below | 61/26 | Reference | 1.04 (0.17, 6.47) | 3.51 (0.50, 24.49) | 0.63 (0.08, 5.01) | 0.822 |
| High school or technical school education | 88/56 | Reference | 0.93 (0.27, 3.22) | 2.68 (0.63, 11.41) | 1.68 (0.42, 6.65) |  |
| College degree or above | 240/177 | Reference | 1.35 (0.59, 3.06) | 1.99 (0.86, 4.63) | 1.66 (0.70, 3.96) |  |
| Dysmenorrhea: Yes | 97/73 | Reference | 3.29 (0.73, 14.80) | 4.34 (0.96, 19.64) | 2.11 (0.50, 8.92) | 0.547 |
| Dysmenorrhea: No | 292/186 | Reference | 1.02 (0.51, 2.04) | 2.06 (0.96, 4.44) | 1.34 (0.64, 2.85) |  |
| Nulliparous | 252/170 | Reference | 1.54 (0.69, 3.44) | 2.07 (0.91, 4.73) | 1.40 (0.61, 3.22) | 0.453 |
| Multiparous | 137/89 | Reference | 0.68 (0.24, 1.91) | 2.63 (0.73, 9.47) | 1.37 (0.44, 4.34) |  |

Abbreviations: BMI, body mass index; CI, confidence interval; FIB, fibrinogen; OR, odds ratio.

Note: ORs were estimated for FIB quartiles within each subgroup. Models were adjusted for the same covariates as the fully adjusted model, except for the stratification variable where applicable. Q1 was used as the reference group unless otherwise indicated. NA indicates that the estimate was not available due to sparse data. Interaction P values were obtained from likelihood ratio tests.

**Supplementary figure**

Figure S1. Exploratory spline curves for APTT, PT, TT, and INR. The solid lines represent the estimated probability of endometriosis, and the shaded areas represent the pointwise 95% confidence intervals. Curves were plotted across the central observed range of each parameter to reduce the influence of sparse extreme values.
